# The genetic control of neocortex volume and covariation with neocortical gene expression in mice

**DOI:** 10.1186/1471-2202-10-44

**Published:** 2009-05-09

**Authors:** Shiv M Gaglani, Lu Lu, Robert W Williams, Glenn D Rosen

**Affiliations:** 1 Division of Behavioral Neurology, Department of Neurology, Beth Israel Deaconess Medical Center, Boston, MA 02215, USA; 2 Center for Neuroscience, Department of Anatomy and Neurobiology, 875 Monroe Ave., Memphis TN 38163, USA

## Abstract

**Background:**

The size of the cerebral cortex varies widely within human populations, and a large portion of this variance is modulated by genetic factors. The discovery and characterization of these genes and their variants can contribute to an understanding of individual differences in brain development, behavior, and disease susceptibility. Here we use unbiased stereological techniques to map quantitative trait loci (QTLs) that modulate the volume of neocortex.

**Results:**

We estimated volumes bilaterally in an expanded set of BXD recombinant inbred strains (n = 56 strains and 223 animals) taken from the Mouse Brain Library . We generated matched microarray data for the cerebral cortex in the same large panel of strains and in parental neonates to efficiently nominate and evaluate candidate genes. Volume of the neocortex varies widely, and is a heritable trait. Genome-wide mapping of this trait revealed two QTLs – one on chromosome (Chr) 6 at 88 ± 5 Mb and another at Chr 11 (41 ± 8 Mb). We generated both neonatal and adult neocortical gene expression databases using microarray technology. Using these databases in combination with other bioinformatic tools we have identified positional candidates on these QTL intervals.

**Conclusion:**

This study is the first to use the expanded set of BXD strains to map neocortical volume, and we found that normal variation of this trait is, at least in part, genetically modulated. These results provide a baseline from which to assess the genetic contribution to regional variation in neocortical volume, as well as other neuroanatomic phenotypes that may contribute to variation in regional volume, such as proliferation, death, and number and packing density of neurons

## BackGround

The cerebral cortex is among the most complicated structures in the brain of mammals, and comprises a large but highly variable fraction of the total brain volume. The volume of the human cortex varies by as much as 60% among normal adults. Much of this variation is genetic [1,2] and has been linked with measures of intelligence [3,4], as well as with differences in susceptibility to disorders including developmental dyslexia [5], anxiety-related personality traits [6], and schizophrenia [7,8]. Dissecting those genetic variants that modulate normal variation of the cerebral cortex could have an impact on our understanding of cortical development, normal function, and the etiology of several pervasive diseases.

In the present study we use BXD recombinant inbred (RI) strains of mice to investigate the genetic basis of normal variation in the size of the neocortex. This genetic reference population, which is both genetically diverse and phenotypically well-characterized, provides an experimental system to test relations and interactions between normal variation among a potentially unlimited variety of traits [9]. Thus, the scientific community has been able use these mice to systematically accumulate a vast amount of morphometric, behavioral, and physiologic data for each strain. This in turn enables scientists with different interests and expertise to test and verify entire systems of traits, their covariance, and their genetic causes [10-13].

Recently, Beatty and Laughlin [14] identified a QTL for neocortical volume on chromosome (Chr) 11 using images of BXD cases taken from the Mouse Brain Library (MBL; ). Dong *et al*. [15] also used the same BXD reference population – and many of the same cases and images from the MBL – and found suggestive QTLs for gray matter volume on Chrs 2, 8, 16, and 19, but not on Chr 11. These studies were constrained by the relatively small number of strains (n = 34) and cases. The recent addition of many new BXD strains and cases to the MBL [16] has significantly increased the utility of this RI set and improved both the power and precision of QTL mapping. In this experiment, we used unbiased stereology to estimate the neocortical volume in both right and left hemispheres in 54 BXD RI lines, and both parental strains. We mapped QTLs modulating neocortical volume to intervals on Chrs 6 and 11. We exploited a variety of bioinformatic resources, as well as our own neonatal and adult databases of gene expression in the neocortex, to identify potential candidate genes within these QTLs.

## Results

### Neocortical volume is highly variable

**Figure 1:**
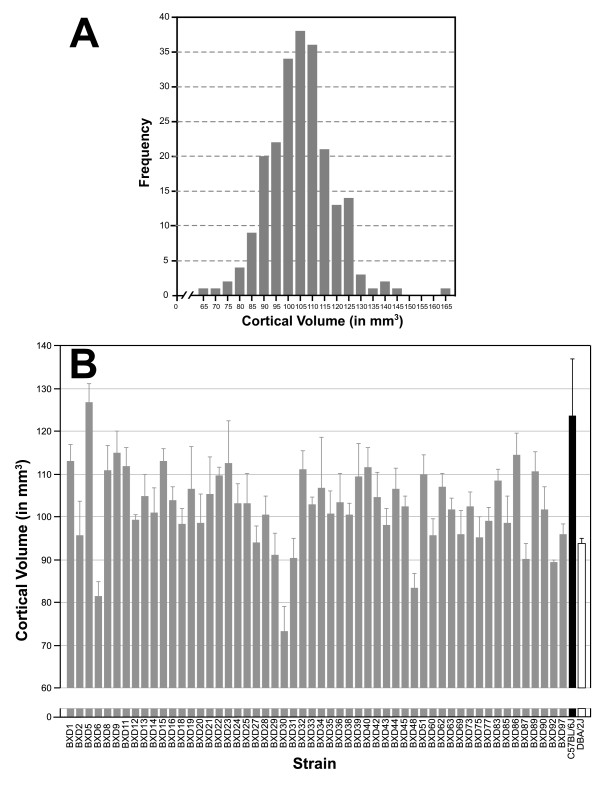
**A. Neocortical volume is normally distributed**. **B**. Mean ± SEM neocortical volume in BXD RI lines (gray bars) and their parental strains, C57BL/6J (black bars) and DBA/2J (white bars).

All estimates of volume are fully corrected for case-by-case differences in shrinkage and should be considered close to the original size of these regions in well-fixed tissue. Bilateral neocortical volume of individual mice has a remarkably wide range – from 61.6 to 162.2 mm^3 ^(mean ± SEM = 102.5 ± 0.9 mm^3^), and is normally distributed (Fig 1A). Strain averages extend from a low of 73.4 ± 4.5 mm^3 ^in BXD30 to a high of 126.9 ± 3.8 mm^3 ^in BXD5 (Fig 1B). The right and left hemisphere neocortical volumes of individual mice ranged from 30.4–82.3 mm^3 ^and from 31.2–79.9 mm^3^, with means of 51.5 ± 0.45 mm^3 ^and 51.1 ± 0.45 mm^3^, respectively.

### There is no significant left/right asymmetry in the neocortex

We computed an asymmetry coefficient *δ*_*asymm *_by subtracting the volume of the left from right hemisphere and dividing by one half of the total volume. To determine whether there were overall asymmetric biases irrespective of direction, we calculated an absolute asymmetry coefficient (*δ*_*abs*_) by taking the absolute value of *δ*_*asymm*_. We computed the distribution of both *δ*_*asymm *_and *δ*_*abs *_for the neocortex (mean ± SEM = -0.007 ± 0.003 and 0.039 ± 0.002 mm^3^, respectively). ANOVA with strain as the independent measure and *δ*_*asymm *_and *δ*_*abs *_as the dependent measures revealed no significant effects of strain (*F*_53,169 _< 1, NS in both cases). These results suggest that there is no significant left/right neocortical asymmetry in this population.

### Neocortical volume is heritable, but asymmetry is not

In order to determine the heritability of neocortical volume, we computed an ANOVA with strain as the independent measure and bilateral volume as the dependent measure. We found a significant effect of strain (*F*_55,167 _= 3.2, *P *< .001). The strain main effect accounts for 51% of the variance, which provides a reasonable upper bound on the fraction of variance that might be explained by additive and epistatic interactions. (Dominance effects cannot be measured using RI strains because there are no heterozygous genotypes or heterozygote phenotypes to analyze.)

There were no significant main effects for *δ*_*asymm*_(*F*_55,167 _< 1, NS) or *δ*_*abs *_(*F*_55,167 _= 1.1; explained variance = 22–27%). These results strongly support the notion that there are significant differences between strains on the volumetric measures, but that strain differences in asymmetry are not apparent.

We also computed heritability (*h*^2^) using inbred strain data and the adjustment method of Hegmann and Possidente [17]. Neocortical volume is a moderately heritable trait (*h*^2 ^= 0.29). The broad sense estimate of heritability, which takes into account the large sample sizes of genetically identical members of RI strains, yielded an *h*^2 ^of 0.67. The heritability factor for *δ*_*asymm *_and *δ*_*abs *_was 0.10 and 0.02, respectively. This low heritability strongly suggests that neither of these traits could be profitably mapped.

### Mapping neocortical volume

**Figure 2:**
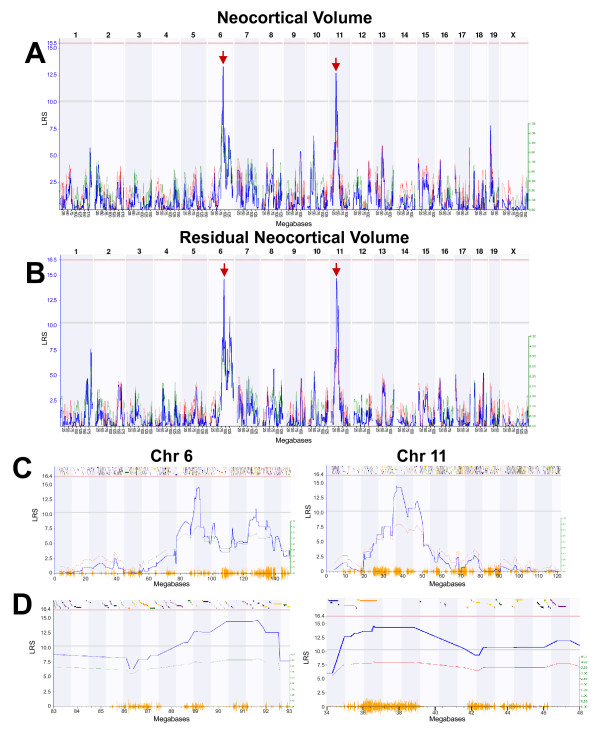
**Mapping neocortical volume**. **A**. Likelihood ratio statistic (LRS) scores for neocortical volume across the entire genome. The x-axis represents the physical map of the chromosome; the y-axis and thick blue line provide the LRS of the association between the trait and the genotypes of markers. The two horizontal lines are the suggestive (blue) and significance (red) thresholds computed using 1000 permutations. There are QTLs (red arrows) on the distal end of Chr 6 and the proximal end of Chr 11. **B**. QTLs for residual neocortical volume (regressing out the effects of age, sex, plane of section, epoch, and non-neocortical brain weight) are identical to those seen in A, but with higher LRS scores. **C**. LRS map of all of Chr 6 (left) and Chr 11 (right) where the peak LRS score can be seen. Orange lines on x-axis represent high density SNP map. Discontinuous track along the top are the genes on this chromosome. **D**. A 10 Mb interval bordering the QTL on Chr 6 (left) and Chr 11 (right).

We mapped bilateral neocortical volume and detected two loci (Fig 2A) with closely matched likelihood ratio statistics (LRS) linkage scores but with opposite allelic effects (that is, *B6 *vs *D2 *alleles contributing to greater volume). The first locus on Chr 6 peaks between 88 and 92 Mb (LRS = 13.3, LOD = 2.9). This region corresponds to human Chr 3 at ≈ 134 Mb (3p25.1). The second locus on Chr 11 peaks between 35 and 40 Mb (LRS = 12.7, LOD = 2.6, corresponding to human Chr 5 at ≈ 158 Mb, 3q35.1).

In order to map variation related to volume of neocortex rather than possible confounding covariates, we performed multiple regression to remove all covariance associated with differences in age, sex, plane of section, strain epoch (the original Taylor BXD set versus the newer UTHSC BXD set; see [16], for an explanation of the history of BXD strains), and non-neocortical brain weight (brain weight – neocortical weight). There were significant effects of non-neocortical brain weight (*F*_1,209 _= 10.8, *P *< .01) and epoch (*F*_1,209 _= 8.2, *P *< .01; Taylor strains = 103.5 ± 1.1 mm^3^; UTHSC strains = 100.3 ± 1.4). Effects of sex (*F*_1,209 _< 1, NS; females = 101.1 ± 1.2 mm^3^; males = 102.6 ± 1.3 mm^3^), plane of section (*F*_1,209 _= 2.1, NS; horizontal = 101.2 ± 1.3 mm^3^; coronal = 103.5 ± 1.2 mm^3^), or age (*F*_1,209 _= < 1, NS) were modest and did not reach statistical significance. We regressed neocortical volume for each subject against age, sex, plane of section, epoch, and non-neocortical brain weight and calculated residuals. The use of all variables, even those that do not reach an alpha level of .05, is warranted and removes that variable as a potential confound in mapping. These residuals were used to compute adjusted strain means.

When we re-mapped the variation in adjusted neocortical volume, we detected the same two loci as the original values (Fig 2B), but with a modest increase in the strength of linkage (Chr 6 at 88 ± 5 Mb LRS = 14.5, LOD = 3.1; Chr 11 at 37 ± 5 Mb LRS = 14.4, LOD = 3.1). The *D2 *allele has a positive effect of about 4 mm^3 ^per allele at the Chr 6 locus, whereas the *B6 *allele has a similar positive effect at the Chr 11 locus (Additional File 1). We did not detect any epistatic interactions that could account for adjusted neocortical volume. Composite interval mapping of neocortical volume did not reveal any significant or consistent secondary loci for this trait.

### Candidate gene analysis

The Chr 6 and Chr 11 loci can be subdivided into large blocks that have common haplotypes in B6, D2, and BXD strains. These blocks are essentially identical by descent (except for any recent mutations) and have low densities of single nucleotide polymorphisms (SNPs). Such regions are less likely to contain polymorphisms that modulate the volume of forebrain derivatives. In contrast, several large blocks have dissimilar haplotypes and much higher densities of SNPs (Figs 2C, D). Genes within these regions have a higher prior probability of containing functional polymorphisms.

#### Chr 11 candidate analysis

We combined data on SNP density with LRS values to rank positional candidate genes. Of ≈ 70 genes in the Chr 11 interval (34–49 Mb), 17 were located in regions that were highly polymorphic (1 to 6 SNPs per kb). Members of this subset are good *positional *candidates, irrespective of any role that they may have in brain development or adult forebrain structure (Additional File 2). An additional 9 candidates were located in regions with low SNP densities but had either missense mutations or SNPs in their promoters or UTRs. The restrictive criteria for inclusion were increased by requiring that strong candidates also have moderate to high expression in the neocortex at some stage of development. To apply this filter, we extracted data on expression of all of these genes in the neocortex from the adult and P1 databases. This subset of 26 genes was ranked using data on missense SNPs in these genes and by evidence of local regulatory variation that controls their own expression – so-called cis-QTLs. Those genes with SNPs in Illumina microarray probes that produced high cis-QTLs were excluded from the analysis. Fifteen genes met these criteria: *Slit3*, *Odz2*, *Gabrg2, Gabra1, Gabrb2, Atp10b, Pttg1, Slu7, Ttc1*, *Ublcp1*, *Ebf1, Adam19, Cyfip2, Thg1l*, and *Itk *(Table 1).

**Table 1:** Candidate genes in QTL interval on Chr 11

**Symbol**	**Gene Description**	**SNP Region**
*Slit3*	slit 3	exons 34, 35
*Odz2*	odz, odd Oz/ten-m 2	exon 9
*Gabrg2*	GABA A receptor, gamma 2	promoter
*Gabra1*	GABA A receptor, alpha 1	Promoter, exon 1 (2×)
*Gabrb2*	GABA A receptor, beta 2	Prom, exons 2 and 10
*Atp10b*	ATPase, class V, type 10B	promoter
*Pttg1*	pituitary tumor-transforming 1	
*Slu7*	SLU7 splicing factor	promoter
*Ttc1*	tetratricopeptide repeat domain 1	promoter, exon 7
*Ublcp1*	ubiquitin-like domain containing CTD phosphatase 1	promoter, exon 1 (3×)
*Ebf1*	early B-cell factor 1	promoter, exon 1
*Thg1l*	tRNA-histidine guanyltransferase 1-like	promoter
*Adam19*	ADAM metallopeptidase domain 19 (meltrin beta)	promoter, exon 23
*Cyfip2*	cytoplasmic FMR1 interacting protein 2	promoter, exon 1 (2×)
*Itk*	IL2-inducible T-cell kinase	exon 1 (2×)

#### Chr 6 candidate analysis

The Chr 6 interval identified here was previously associated with striatal volume, and the identification of candidate genes (using the criteria outlined above), was identical to those recently described [18]. A subset of 10 genes met all the criteria: *Htra2*, *Tia1*, *Mxd1*, *Anxa4*, *Aak1*, *Nfu1*, *Nup210*, *Hdac11*, *Fbln2*, and *Slc25a26 *(Table 2).

**Table 2:** Candidate genes in QTL interval on Chr 6

**Symbol**	**Gene Description**	**SNP Region**
*Htra2*	HtrA serine peptidase 2	exon 8
*Tia1*	T-cell restricted intracellular antigen 1	promoter (2×), 3' UTR (2×)
*Mxd1*	MAX dimerization protein 1	exon 6
*Anxa4*	annexin A4	exon 13
*Aak1*	AP2-associated kinase 1	exon 15
*Nfu1*	NFU1 iron-sulfur cluster scaffold homolog	exon 2
*Nup210*	nucleoporin 210	exons 10,17,19,21
*Hdac11*	histone deacetylase 11	promoter (× 5)
*Fbln2*	fibulin 2	exons 2 (× 3), 6
*Slc25a26*	solute carrier family 25 (mitochondrial carrier, phosphate carrier), member 26	promoter

#### Gene Expression correlations with neocortical volume

**Figure 3:**
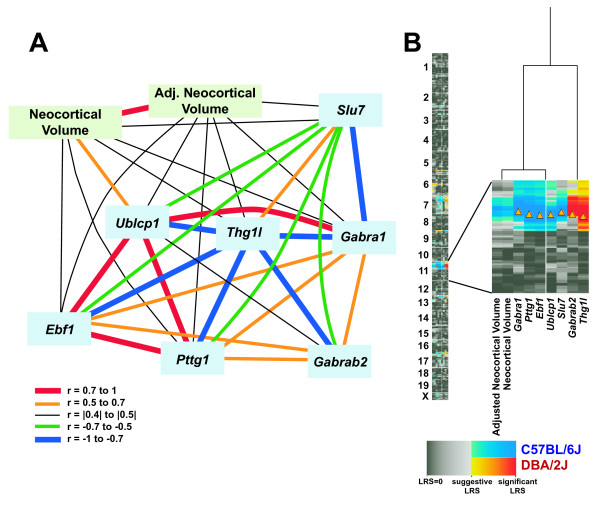
**Covariation of neocortical volume with candidate genes on Chr 11**. **A**. Correlations among expression levels across the BXD RI set of positional candidate genes (blue squares) on Chr 11 and neocortical volume (green squares). Red and orange lines indicate Pearson correlations of > +0.7 and +0.5 to +0.7, respectively. Blue and green lines indicate correlations of < -0.7 and -0.5 to -0.7, respectively. Black lines have correlations between |0.4| and |0.5|. Line thickness varies with strength of correlation. Neocortical volume correlates with these transcripts, and there are strong intercorrelations among the transcripts themselves. **B**. QTL cluster map of transcripts and phenotypes listed in A. This is a "hot map" of LRS scores, with red indicating modulation by DBA/2J alleles, and blue indicating modulation by C57BL/6J alleles. The enlarged portion of Chr 11 indicates that the 7 transcripts are cis-acting QTLs, i.e., genes that modulated their own expression (orange triangles indicate genetic location of the transcript).

We asked which subset of the positional candidates at both QTLs also had differences in expression across BXD strains that co-varied well with our trait. We produced a correlation matrix based on the subset of 47 strains for which we have data of all types. This matrix was then thresholded at a Pearson r = |0.4| and plotted as a graph. There were 7 genes – *Gabra1, Gabrb2, Pttg1, Slu7, Ublcp1, Ebf1*, and *Thg1l *– that co-varied with neocortical volume on Chr 11 (Fig 3). We further found confirmatory evidence from the Allen Brain Atlas and GENSAT that these genes are expressed either in the adult brain, the developing brain, or both. In marked contrast, none of the Chr 6 gene expression profiles co-vary with neocortical volume.

#### Trait correlation analysis

One advantage of employing the BXD RI set is the ability to test for covariation with other phenotypes that have also been studied in this population. We correlated neocortical volume (adjusted and non-adjusted) with the BXD Published Phenotypes Database of GN, which contains a large number of behavioral, anatomic, and physiologic phenotypes gathered from BXD RI sets. Alpha levels were adjusted after permuting neocortical volume and adjusted neocortical volume 20 times each, determining the top 50 correlations with the BXD Published Phenotypes Database for each permutation, and determining the computed alpha level of the top 5% of all correlations. From this analysis, it was determined that computed correlations by GN with *P *< .001 were significant at an adjusted alpha level of .05.

Both neocortical and adjusted neocortical volume correlated highly with a large number of morphological traits, including volume of dorsal thalamus, dorsal striatum, lateral geniculate nucleus, hippocampus, basolateral amygdala, fimbria, trigeminal tract, nucleus accumbens, and corpus callosum. There were no significant correlations with non-morphological traits.

## Discussion

We systematically explored sources of genetic variation in the size of the neocortex. Using unbiased stereological estimations of this trait in the BXD RI genetic reference population, we found that neocortical volume is highly heritable and variable. There was a 2.6-fold difference between the neocortical volume of the smallest and largest individuals in this study, and a 1.7-fold difference when strain means were compared. The heritability of this trait is consistent with that reported for similar neuroanatomic traits including olfactory bulb, hippocampus, striatum, neocortex, and thalamus [12,14,19-24]. Even with a large sample size and a powerful paired *t*-test design, we did not find any significant asymmetry between the left and right hemispheres of mice. Neocortical volume was associated with QTLs on Chrs 6 and 11. Large microarray data sets were used to efficiently nominate small numbers of viable candidate genes, one or more of which may modulate the volume of the neocortex, and these results supported candidates on the Chr 11 interval as modulating neocortical volume.

### There is a lack of anatomic asymmetry in the neocortex

Because previous research has found evidence of volumetric asymmetry in the rodent brain [25-31], we attempted to assess this variable in the BXD RI set. We found that there was little evidence of asymmetry in the neocortex. This was true both for measures that assessed directional (right vs. left) asymmetry and those that assessed magnitude (without considering directionality). These results are consistent with previous reports that failed to reveal neocortical asymmetries in the mouse brain [32]. The results reported here suggest that there are no population level anatomic asymmetries in the neocortex, but do not discount the possibility that anatomic asymmetries at the individual level may correlate with functional asymmetries.

### QTLs for neocortical volume

Using images from the MBL, Beatty and Laughlin [14] reported significant QTLs for neocortical volume on Chr 11 and for non-neocortical brain volume on Chr 19. A more recent study, which again used images from the MBL [15], did not find any statistically significant QTLs for gray matter volume, although there were suggestive QTLs on Chrs 2, 8, 16, and 19.

We find some support in this study for the Chr 11 QTL reported by Beatty and Laughlin [14]. Although the Chr 11 interval identified by Beatty and Laughlin is more proximal than that described in the current study, the intervals do overlap. In some ways, this was not surprising given that both Beatty and Laughlin and we analyzed brains from the MBL. Yet there were a number of methodological differences that could have worked against this. Our estimations of cerebral volume were performed using unbiased stereology on histologic tissue that was corrected for section thickness, whereas Beatty and Laughlin used planimetric techniques on images from the MBL to estimate volume. Despite these differences in methodology, there was a highly significant correlation (*r *= 0.78, *P *< .001) between the neocortical volume estimates of Beatty and Laughlin and our own for the 36 strains common to both studies, and the correlation between the regressed cerebral volume estimates – the variable used to map these traits – is also quite high (*r *= 0.68,*P *< .001). This was encouraging given that we regressed out the effects of brain weight (minus the weight of the cortex itself), age, plane of section, epoch, and sex, whereas Beatty and Laughlin regressed out brain weight and logarithm of age only.

There were differences, however, between our results and those published previously. None of the early data sets detect a QTL on Chr 6, and we find no support for any of the QTLs identified by Dong *et al*. [15]. The most obvious difference among these studies is the inclusion of the new BXD strains [16]. QTL detection and map precision improves with number of strains, whereas increasing the number of subjects within a strain has only marginal effects [33,34]. We have increased the number of strains by over 50% (34 to 54) in this experiment, and it is likely that our ability to detect QTLs that modulate a smaller percent of the variance of neocortical volume is improved.

### Positional candidate genes

Before considering positional candidate genes, it may be useful to review factors that are likely to impact normal variation in brain volume. Cerebral cortical volume can be modified by a variety of developmental factors, as well as those that appear during adulthood. Thus, genes modulating cell proliferation, differentiation, and/or death may contribute to the variation in cerebral cortical volume reported here. In adulthood, cerebral cortical volume can be modified by neurodegenerative changes, some of which may be under genetic control. In the current experiment, age was not a significant predictor of cerebral cortical volume, and none of the mice would be qualified as senescent. When considering potential candidate genes, we therefore concentrated our efforts on those genes known to modulate factors in brain development.

We have recently reported a QTL modulating striatal volume on the same interval on Chr 6 as that in the current report [18]. A detailed analysis of the genes lying within that chromosome (see Table 2) identified a number of potential candidate genes. Analysis of gene expression strain distribution profiles revealed that a number of these genes correlated well with striatal volume. In contrast, none correlated with cerebral cortical volume in the current study. In a complementary manner, none of the Chr 11 candidates reported here correlated with striatal volume.

There are a number of genes in the Chr 11 interval whose functions suggest that they could play a role in modulating neocortical volume. *Slit3 *is a well-characterized gene that is part of the Slit family of ligands for the Robo receptors [35], and which are important for neuronal migration, axon outgrowth, and dendritic development [e.g., [36]]. Moreover, it has recently been demonstrated that this gene is upregulated in the caudal ganglionic eminence, which gives rise to neocortical GABAergic neurons [37]. This is particularly intriguing given the inclusion of three GABA receptor subtype genes (*Gabrg2*, *Gabra1*, and *Gabrb2*) in the QTL interval. *Odz2 *is expressed in the mouse brain quite early during development [38], although its specific role is not yet known. *Cyfip2 *has been shown to be an interactor with FMRP (fragile × mental retardation protein), which has been implicated in Fragile × syndrome, and is highly expressed in neurons throughout the brain [39].

Of the candidate genes on Chr 11, we found seven whose expression levels were highly correlated with neocortical volume. All are strong cis-QTLs; that is, a sequence variant in or very close to the gene itself controls its expression (Fig 3). Many of these genes have been documented to have roles during brain development. GABA receptors (see above) are obvious candidates given their roles in brain development in general and neocortical functioning in specific [e.g., [40]]. *Pttg1 *is upregulated during development of the mouse telencephalon [41]. Likewise, *Ublcp1 *is upregulated in fast growing or tumor tissues perhaps through its activity as a regulator of the phosphorylation state of RNA Polymerase II [42]. The transcription factor *Ebf1 *plays a pivotal role in ensuring that a set of thalamic axons reaches the neocortex as well as in the development of striatal neurons [43,44]. Though the last two transcripts, *Slu7 *and *Thg1l*, have not yet been implicated in neural growth, their molecular functions suggest a developmental role. The homolog of *Slu7 *is well characterized in *S. cerevisae *as essential during the second catalytic step in the pre-mRNA splicing process [45]. Additionally, *Thg1l *is important for tRNA priming for protein translation [46].

### Future Directions

Mechanisms that control numbers of neurons and glial cells assigned during development to the neocortex are crucial in understand human behavior, human evolution, and the basis of individual differences. This study can be considered an essential step in a more refined regional genetic anatomy of the several dozen distinct cytoarchitectonic regions that together make up the entire neocortex. The cellular demographics of the neocortex are a strong determinant of functional repertoire. This is most obvious when comparisons are made at a coarse taxonomic level comparing, for example, the massive auditory cortex of echo-locating bats and the complex, highly evolved whisker fields of mice and rats, and the expanded prefrontal cortical regions of humans. But even within a species, pronounced differences in cytoarchitecture are associated with function [47-49]. Now that we have a baseline, we can begin the more painstaking genetic studies of discrete regions of the mouse neocortex and determine if there are QTLs with smaller but regionally intense effects on, for example, barrel cortex, primary visual cortex, or temporal regions.

A number of research strategies that can further refine the QTL interval are available. Higher density maps of the B6 and D2 parental strains will allow a more refined screen for sequence variants. Knockouts of the candidate genes are another potentially productive avenue of investigation. Although none of the Chr 11 candidates have been knocked out, *Pttg1*, *Ebf1*, *Ublcp1*, *Slu7*, *Thg1l*, and *Gabrb2 *have been targeted by the Knockout Mouse Project . Additionally, there are a number of forward genetic methods that can be employed to confirm our results. The interval identified here using the BXD RI line could be confirmed in other recombinant inbred lines, including the AXB, BXA, and LXS. The Collaborative Cross – an 8-strain cross to generate hundreds of RI lines, each with more recombinations than existing RI strains – will enhance precision of QTL mapping [50-52]. Recombinant Inbred Intercross (RIX) schemes – whereby selective generation of F1 progeny from recombinant inbred parents allows for the examination of known heterozygous and homozygous intervals – extends the number of genomes that can be phenotyped by n(n-1)/2, where n is the number of original recombinant inbred strains (in our case, 54). The RIX lines have known genomes and quantifiable phenotypes and, therefore, will contribute to mapping traits to their genetic determinants [53].

## Conclusion

This study is the first to use the expanded set of BXD strains to map neocortical volume. With the enhanced precision garnered from the addition of over 20 strains, we found that neocortical volume is a heritable trait with large inter-strain variability. By quantifying cerebral cortical volume for both the right and left hemispheres, we were able to conclude that asymmetry is most likely not genetically modulated, at least in this group of inbred mice. We identified two QTLs, one on Chr 6 and another on Chr 11, that are likely to contain gene variants that modulate neocortical volume. From correlational analysis to gene expression data, we identified a number of candidates on the Chr 11 interval that may modulate this trait. Future research exploiting this expanded BXD genetic reference population will allow us to further dissect other important neuroanatomic phenotypes that may contribute to variation in regional volume, such as proliferation, death, and number and packing density of neurons.

## Methods

All experiments were approved by the Institutional Animal Care and Use Committees at Beth Israel Deaconess Medical Center and University of Tennessee Health Science Center.

### Subjects

All histologic data for this study were obtained from The Mouse Brain Library (MBL) – a physical and Internet resource that contains high-resolution images of histologically processed slides from over 2900 adult mouse brains  with roughly balanced numbers of male and female specimens [9]. The ages ranged from 21–694 days of age (mean ± SEM = 103 ± 5), with most of the cases ranging from 50 – 120 days. Mice were obtained from either the Jackson Laboratory (Bar Harbor, ME) or the University of Tennessee Health Science Center (UTHSC) as detailed previously [24]. All procedures were approved by institutional animal care and use committees and conform to NIH guidelines for humane treatment of animals. Mice were deeply anesthetized with Avertin (0.8 ml i.p.) and transcardially perfused with 0.9%saline, followed by fixative (1.25% glutaraldehyde/1.0% paraformaldehyde in phosphate buffer), and their brains removed and weighed. After variable post-fixation times, the brains were embedded in 12% celloidin and sliced in either a coronal or horizontal plane at a width of approximately 30 μm. Actual section thickness was determined by direct examination of 10 sections for each brain using an ×100 oil immersion objective and a z-axis micrometer.

### Estimation of neocortical volume

The volume of the neocortex was estimated in 223 mice (114 female and 109 male) from 54 BXD RI strains and the two parental strains (C57BL/6J and DBA/2J, abbreviated B6 and D2, respectively) by one of us (SMG) using a computer controlled microscope (Nikon E800, Nikon, Inc., Melville, NY) and Stereo Investigator (MBF Biosciences, Williston, VT). For most strains, 4 mice were used with the exception of BXD2 (N = 3), BXD15 (N = 5), and BXD83 (N = 3). Neocortex was parcellated by the criteria of the Allen Brain Atlas (mouse.brain-map.org), and includes agranular insular (AId, AIv, AIp), auditory (AUDv, AUDd, AUDv), cingulate (ACAd, ACAv), ectorhinal (ECT), gustatory (GU), infralimbic (ILA), motor (MOs, MOp), orbital (ORBl, ORBvl, ORBm), parietal (PPLp), prelimbic (PL), perirhinal (PERI), retrosplenial (RSPv, RSPd, RSPagl), somatosensory (SSp, SSs), temporal (TEa), visceral (VISC), and visual (VISam, VISp, VISal) cortices. Volume was estimated by point counting using Cavalieri's method. Grid spacing for horizontal (N = 110) and coronal (N = 113) sections was 800 μm and 400 μm, respectively. For each brain, two interleaved 1-in-10 series of sections were examined, representing every fifth section. For horizontal sections, volume was estimated by measuring every tenth section on each slide, whereas every thirtieth section was measured for coronal sections (six to ten sections per slide). Volume was independently estimated for both the right and left hemispheres, and the sum of these measures estimated total neocortical volume. In cases where there were missing or damaged sections, a piece-wise parabolic estimation was used [54]. Final volume estimates were individually corrected for histological shrinkage by determining the previously computed ratio between the brain volume at fixation (brain weight) and that after processing. Neocortical volume was blindly re-measured on 10 slides to assess intra-observer reliability. The experimenter was blind with respect to strain and sex.

### Measurement error

Intra-observer reliability was high for estimation of neocortical volume. The percentage difference between the original and repeated volume estimations ranged from 0–5% and the average difference was 0.42%. A correlation coefficient between the two measurements was highly significant (*r *= 0.99) indicating that technical error at this level of the analysis contributes little to case variation or strain variation. A paired *t*-test confirmed that the difference between the first and second estimations was not significant (*t *< 1, NS).

### Analysis

Data were analyzed using standard ANOVA and multiple regression techniques (JMP, SAS Institute, Cary, NC). QTL analysis was performed using the WebQTL module of GeneNetwork (GN, ). This online resource includes all known morphometric data for the BXD strains, several neocortical transcriptome data sets, high density marker maps based on approximately 3795 fully informative markers distributed on all chromosomes except Chr Y [55], and a database containing ≈ 8.3 million SNPs taken from dbSNP [56]. WebQTL incorporates three common mapping methods: (1) simple interval mapping, (2) composite interval mapping, and (3) a scan for two-locus epistatic interactions [57].

### Array data

We exploited an expression data set available at  that was generated with support from the CHDI Foundation. This data set estimated steady-state mRNA levels in the neocortex of 73 strains of adult (P60 ± 5) mice, of which 44 are common to the morphometric data described here. Strains for which we have morphometric data but no array data include BXD22, BXD24, BXD25, BXD30, BXD35, BXD48, BXD63, BXD83, BXD85 and BXD92. We specifically used the GeneNetwork database named "HQF BXD Neocortex ILM6.1 (Feb08) RankInv," which can be accessed in the main search page by selecting Species = Mouse, Group = BXD, Type = Neocortex mRNA. Data were generated using the Illumina Sentrix Mouse-6.1 microarray. This array estimates expression for a great majority of mouse genes with confirmed protein products and consists of sets of ≈ 46,000 unique 50-nucleotide-long probe sequences. Like other array data in GeneNetwork [10], the original Illumina bead array data (rank invariant transform) were logged and re-centered to a mean of 8 units and a standard deviation of 2 units – essentially a *Z *transform of the data. For complete metadata on the adult neocortex data set, including quality control procedures, error-checking, and normalization see .

Comparing adult morphometric data with adult cortical expression data is potentially insufficient since it is possible that the transient expression of a small number of genes during development may leave a lasting imprint on differences in volume maturity [but see [58]]. For this reason, we generated a companion expression data set of neonatal (P1) neocortical expression data for the two parental strains, B6 and D2 (NIH Neuroscience Microarray Consortium , rosen-illu-mouse- 588443). Data were generated using the Illumina MouseWG-6 v2 Expression Beadchip. We have exploited this companion developmental data to 1) test whether genes with expression differences that co-vary with neocortical volume at maturity are also expressed during a key stage of development in the neocortex, and 2) to test whether any genes in QTL intervals have high expression only during development.

All genome coordinates in this paper are given using the mouse genome assembly of February 2006 (UCSC Genome Browser release mm8, NCBI Build 36). These position coordinates differ slightly (usually less than 1–3 Mb) from mm9 and NCBI Build 37.1.

### Correlation analysis

To evaluate candidate genes and to study other molecular co-variates of neocortical volume we correlated our estimated volumes with the P1 and P60 neocortical transcriptome data sets. Co-variation networks were constructed using on-line tools in GN. We additionally correlated our neocortical volume data set with a database of over 1000 previously published and unpublished BXD traits .

### On-line data access

Phenotypes for the BXD strains generated as part of this study are all available at Genenetwork.org using the accession numbers:

Neocortical volume (mm^3^): GN BXD Phenotypes Trait ID: 10995

Neocortical volume adjusted (mm^3^): GN BXD Phenotypes Trait ID: 10997

Cerebral cortex volume (mm^3^, Beatty and Laughlin, 2006): GN BXD Phenotypes Trait ID: 10992

Neocortex expression data exploited in this study are directly accessible at  (adult) or arrayconsortium.tgen.org (P1 data set).

## Abbreviations

B6: C57BL/6J; Chr: Chromosome; D2: DBA/2J; GN: GeneNetwork; MBL: Mouse Brain Library; QTL: Quantitative Trait Locus; SNP: single nucleotide polymorphism.

## Authors' contributions

SMG was involved in the design or the experiment, carried out the data acquisition, participated in the data analysis and in the drafting of the manuscript. LL provided the animals used to generate portions of the adult transcriptome database and participated in editing the manuscript. RWW curated the GeneNetwork databases, participated in the data analysis and in the editing of the manuscript. GDR conceived the experiment, supervised the data analysis and drafting of the manuscript. All authors read and approved the final manuscript.

## Supplementary Material

Additional file 1 **Mean neocortical volume arranged by magnitude**. Mean ± SEM neocortical volume in BXD RI lines and their parental strains, C57BL/6J and DBA/2J (arrows), arranged by magnitude of volume. Allelic inheritance at the Chr 11 QTL is indicated by color, blue = D allele, red = B allele. Click here for file

Additional file 2 **Initial candidate genes in Chr 11 interval**. Table denoting candidate genes in the Chr 11 based on the initial screen. Click here for file
