# Supplementary figures and images for: The genetic control of neocortex volume and covariation with neocortical gene expression in mice
Source: BMC Neurosci. 2009 May 9;10:44. doi: 10.1186/1471-2202-10-44 (PMC2685397; doi:10.1186/1471-2202-10-44)

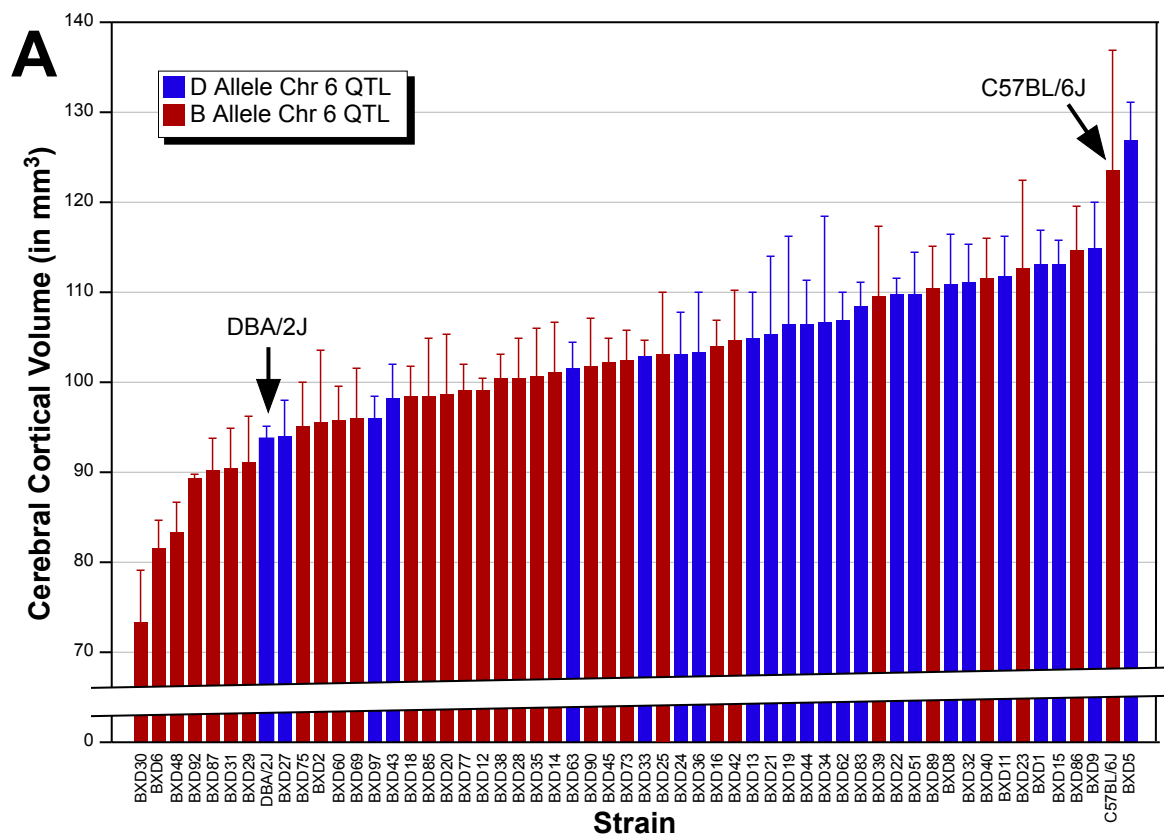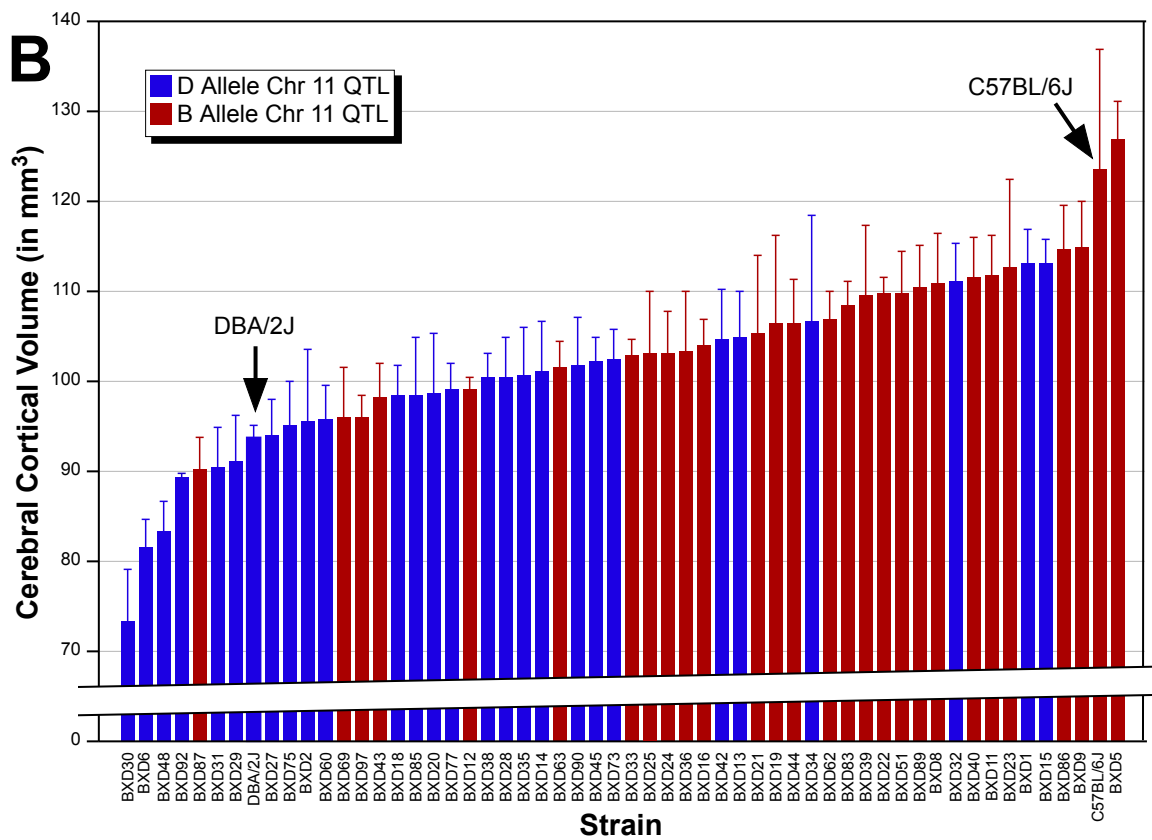

Supplement: Additional file 1 — Mean neocortical volume arranged by magnitude. Mean ± SEM neocortical volume in BXD RI lines and their parental strains, C57BL/6J and DBA/2J (arrows), arranged by magnitude of volume. Allelic inheritance at the Chr 11 QTL is indicated by color, blue = D allele, red = B allele. [file 1471-2202-10-44-S1.pdf]
