# Supplementary material for: The genetic control of neocortex volume and covariation with neocortical gene expression in mice
Source: BMC Neurosci. 2009 May 9;10:44. doi: 10.1186/1471-2202-10-44 (PMC2685397; doi:10.1186/1471-2202-10-44)
Supplement: Additional file 2 — Initial candidate genes in Chr 11 interval. Table denoting candidate genes in the Chr 11 based on the initial screen. [file 1471-2202-10-44-S2.doc]

|  | **SNP** |  |  |  |  |
| --- | --- | --- | --- | --- | --- |
|  | **Density** | **Mb Start** | **Expression** | |  |
| **Gene Symbol** | **(SNP/Kb)** | **(mm^8^)** | **P1** | **P60** | **Max cis** |
| *Dock2* | 0.185858 | 34.126863 | 7.16 | 7.45 | <1 |
| *EG574403* | 0.176223 | 34.214821 | 8.18 | 8.00 | <1 |
| *Slit3* | 1.250735 | 34.96488 | 7.65 | 11.93 | ≈2.5 |
| *Odz2* | 3.778898 | 35.850091 | 11.64 | 12.08 | ≈2 |
| *Mat2b* | 0 | 40.522738 | 9.24 | 8.30 | ≈2 |
| *Gabrg2* | 1.576186 | 41.755279 | 12.18 | 11.44 | 14.4 |
| *Gabra1* | 2.584984 | 41.974902 | 7.77 | 12.81 | 30.4* |
| *Gabrb2* | 2.513684 | 42.263179 | 8.95 | 8.31 | 39.4* |
| *ENSMUSG00000055* | 3.913894 | 42.879168 |  |  |  |
| *Atp10b* | 1.396685 | 42.991319 | 7.62 | 7.65 | 13.2 |
| *Pttg1* | 0.349834 | 43.263687 | 6.82 | 8.79 | 134.1 |
| *Slu7* | 3.929549 | 43.277153 | 10.57 | 10.89 | 40.56* |
| *C1qtnf2* | 1.804214 | 43.317766 | 8.82 | 7.51 | ≈6 |
| *Ttc1* | 0.22263 | 43.573429 | 12.24 | 12.16 | ≈8 |
| *Il12b* | 1.504837 | 44.243485 | 6.74 | 6.71 | <1 |
| *Ublcp1* | 4.100008 | 44.297997 |  | 10.31 | 49.6* |
| *Rnf145* | 2.599188 | 44.362386 |  |  |  |
| *Ebf1/9830166E18Rik* | 1.369378 | 44.461556 | 8.93 | 8.56 | 83.3* |
| *Thg1l* | 2.588203 | 45.791515 |  | 8.31 | 149.8* |
| *Adam19* | 0.197036 | 45.899409 | 10.21 | 9.87 | 21.6* |
| *Cyfip2* | 1.038036 | 46.03728 | 9.77 | 13.60 | ≈8 |
| *Itk* | 2.283814 | 46.168572 | 7.03 | 6.91 | 9.3 |
| *EG432555* | 0.271444 | 48.731623 |  |  |  |
| *OTTMUSG00000005523* | 0.488845 | 48.748403 |  |  |  |

*P < .01. Max cis = maximal cis-QTL. Expression is value derived from microarray. All data in microarray is log base 2 transformed and normalized with a mean of 8 and a standard deviation of 2.
